# Development and validation of brain target controlled infusion of propofol in mice

**DOI:** 10.1371/journal.pone.0194949

**Published:** 2018-04-23

**Authors:** Brenna P. Shortal, Sarah L. Reitz, Adeeti Aggarwal, Qing C. Meng, Andrew R. McKinstry-Wu, Max B. Kelz, Alex Proekt

**Affiliations:** 1 Department of Neuroscience, Perelman School of Medicine, University of Pennsylvania, Philadelphia, Pennsylvania, United States of America; 2 Department of Anesthesiology and Critical Care, Perelman School of Medicine, University of Pennsylvania, Philadelphia, Pennsylvania, United States of America; Scuola Superiore Sant'Anna, ITALY

## Abstract

Mechanisms through which anesthetics disrupt neuronal activity are incompletely understood. In order to study anesthetic mechanisms in the intact brain, tight control over anesthetic pharmacology in a genetically and neurophysiologically accessible animal model is essential. Here, we developed a pharmacokinetic model that quantitatively describes propofol distribution into and elimination out of the brain. To develop the model, we used jugular venous catheters to infuse propofol in mice and measured propofol concentration in serial timed brain and blood samples using high performance liquid chromatography (HPLC). We then used adaptive fitting procedures to find parameters of a three compartment pharmacokinetic model such that all measurements collected in the blood and in the brain across different infusion schemes are fit by a single model. The purpose of the model was to develop target controlled infusion (TCI) capable of maintaining constant brain propofol concentration at the desired level. We validated the model for two different targeted concentrations in independent cohorts of experiments not used for model fitting. The predictions made by the model were unbiased, and the measured brain concentration was indistinguishable from the targeted concentration. We also verified that at the targeted concentration, state of anesthesia evidenced by slowing of the electroencephalogram and behavioral unresponsiveness was attained. Thus, we developed a useful tool for performing experiments necessitating use of anesthetics and for the investigation of mechanisms of action of propofol in mice.

## Introduction

Millions of people receive general anesthesia each year [1]. Yet, the mechanisms by which anesthetics induce reversible loss of consciousness remain incompletely understood [2]. While numerous receptors and binding sites for anesthetics have been identified and characterized, [3] the processes through which these molecular level events lead to changes in patterns of activity of neuronal networks are currently unknown.

One intriguing fact about the effect of anesthetics is that entry into the anesthetized state (induction) occurs at a consistently higher anesthetic concentration than exit from the anesthetized state (emergence). This neural inertia has been demonstrated for volatile anesthetics in *Drosophila*[4] and mouse [5]. Some evidence for neural inertia also exists in humans [6]. Several mutations in *Drosophila* change hysteresis by preferentially affecting either induction or emergence [4]. Interference with the orexinergic [7] and noradrenergic [5] signaling in the mouse brain preferentially affects emergence from anesthesia while leaving induction relatively spared. These data suggest that anesthetic hysteresis is unlikely due to pharmacokinetic factors alone. Rather, it implies that there may not be a simple one-to-one mapping between brain anesthetic concentration and activity of neuronal networks that mediate arousal. This has been hypothesized on the basis of mathematical modeling of cortical networks [8–10]. Consistent with these theoretical results, recordings of local field potentials from the cortex and thalamus of rats maintained on isoflurane revealed that, even when the anesthetic concentration is fixed, brain activity fluctuates abruptly among several quasi-stationary activity patterns [11]. Altogether these findings suggest that intrinsic neuronal dynamics complicate the understanding of anesthetic effects solely in terms of concentration-response relationships.

Studies of hysteresis and neuronal dynamics under anesthesia have thus far focused on volatile anesthetics. One reason for this is that no reliable target-controlled infusion (TCI) model has been able to maintain a fixed concentration of intravenous anesthetic in the brain of a neurophysiologically tractable animal model system. In humans, pharmacokinetics of plasma propofol concentration have been extensively studied, and, as a result, TCI for propofol and other intravenous agents has been available for years [12–15]. Yet, because brain measurements of drug concentration are not available in humans, pharmacokinetic (PK) models assume a hypothetical “effect site” compartment. It is furthermore assumed that there is a one-to-one relationship between the concentration of the drug at the effect site and features of neuronal activity measured by EEG [14–16].

The introduction of TCI in humans allowed for more robust investigation of brain activity under anesthesia [17–20], Human experiments are necessarily limited, however, because direct recording of brain activity requires invasive techniques. Furthermore, the assumption of the effect site concentration is not readily testable in humans. Therefore, there is great need for the development of pharmacokinetic models and TCI in animal models that can be readily used for invasive recordings of brain activity in a genetically tractable mammal.

In addition to the utility of robust TCI for anesthetic mechanisms research, there is growing realization in the basic neuroscience community that the type of anesthetic can have a very significant impact on the observed neuronal responses [21]. Because TCI is not readily available for laboratory animals, anesthetic concentration in the brain is not easily controlled in most studies e.g. [22–27]. This hampers direct comparisons across different studies.

Thus, here we set out to develop a pharmacokinetic model for propofol in mice. The ultimate goal of this exercise is to be able to hold brain concentration of propofol fixed at the desired level. Furthermore, we developed Matlab software that interfaces our model with a syringe pump and continuously adjusts infusion rate to compensate for the distribution and elimination of propofol in order to maintain brain propofol concentration constant.

## Materials and methods

### Animals

All experiments in this study were approved by Institutional Animal Care and Use Committee at the University of Pennsylvania and were conducted in accordance with the National Institutes of Health guidelines. All experiments were performed using adult (2.90 ± 0.45 months) male C57BL/6 mice (Jackson Laboratories). Mice were housed under a reverse 12:12 h, light:dark cycle. Mice were provided with food and water *ad libitum*.

Altogether, data from 21 mice (241 samples: 193 brain, 48 blood) were used to fit the model parameters. The model validation was performed on a separate group of 10 mice (80 samples: 68 brain, 12 blood). Thus, 321 samples (261 brain, 60 blood) collected from 31 mice were used in this study. Neurophysiological confirmation of anesthetic depth was performed in two final mice.

### Surgery

All surgery was performed under aseptic conditions. Each animal was weighed immediately prior to surgery. All mice were within a normal body-weight range (27.4 ± 1.5 g) for adult mice. After weighing, anesthesia was induced with 2.5% isoflurane in an induction chamber. Once loss of righting reflex was established, the animal was placed on its back and provided isoflurane anesthesia through a nose cone. Isoflurane concentration was adjusted such that no response to toe pinch was elicited (~ 1%). An animal’s core-body temperature was maintained at 37 (± 0.5) °C using a temperature controller with core-body temperature monitoring (TC-1000 Temperature Controller, CWE, Incorporated, Ardmore, PA, USA). Jugular cannulation was performed using a technique similar to that described previously [28]. Once the jugular catheter was in place, the mouse was turned to its ventral surface and placed into a stereotaxic frame (Kopf Instruments). The scalp was retracted permitting maximum exposure of the surface of the skull. The bone was cleaned and dried before bilateral craniotomies were performed using a dental drill. Craniotomies extended from bregma to lambda and from near midline to as far laterally as possible. Finally, a durotomy was performed on each side to expose the surface of the cortex. Subsequently, gelfoam (Pfizer) was placed on the exposed cortex to prevent the tissue from dehydrating.

### Propofol infusion

Prior to the initiation of propofol infusion, isoflurane was lowered to 0.2%. This is a sub-anesthetic dose at which 100% of mice regain righting reflex [5]. Even at 1% isoflurane, no significant deviation from baseline hemodynamics are observed [29]. The jugular catheter was connected to an infusion line pre-filled with propofol (10 mg⋅mL^-1^, Fresenius Kabi USA, LLC, Lake Zurich, IL, USA) and a syringe pump (Pump 11 Elite, Harvard Apparatus, Holliston, MA, USA). Once the connection was established, the syringe pump was used to flush propofol and assure that all of the saline was purged from the jugular catheter. In order to minimize the effect of inadvertent introduction of flushed propofol into the circulation on subsequent propofol measurements, 10 minutes elapsed between clearing the catheter and the beginning of the infusion. Infusions were driven using a custom program written in MATLAB (Mathworks, R2014a) using USB communication between the pump and a computer (MAC Mini, Apple Incorporated, Cupertino, CA, USA).

### Sampling

**Fig 1:**
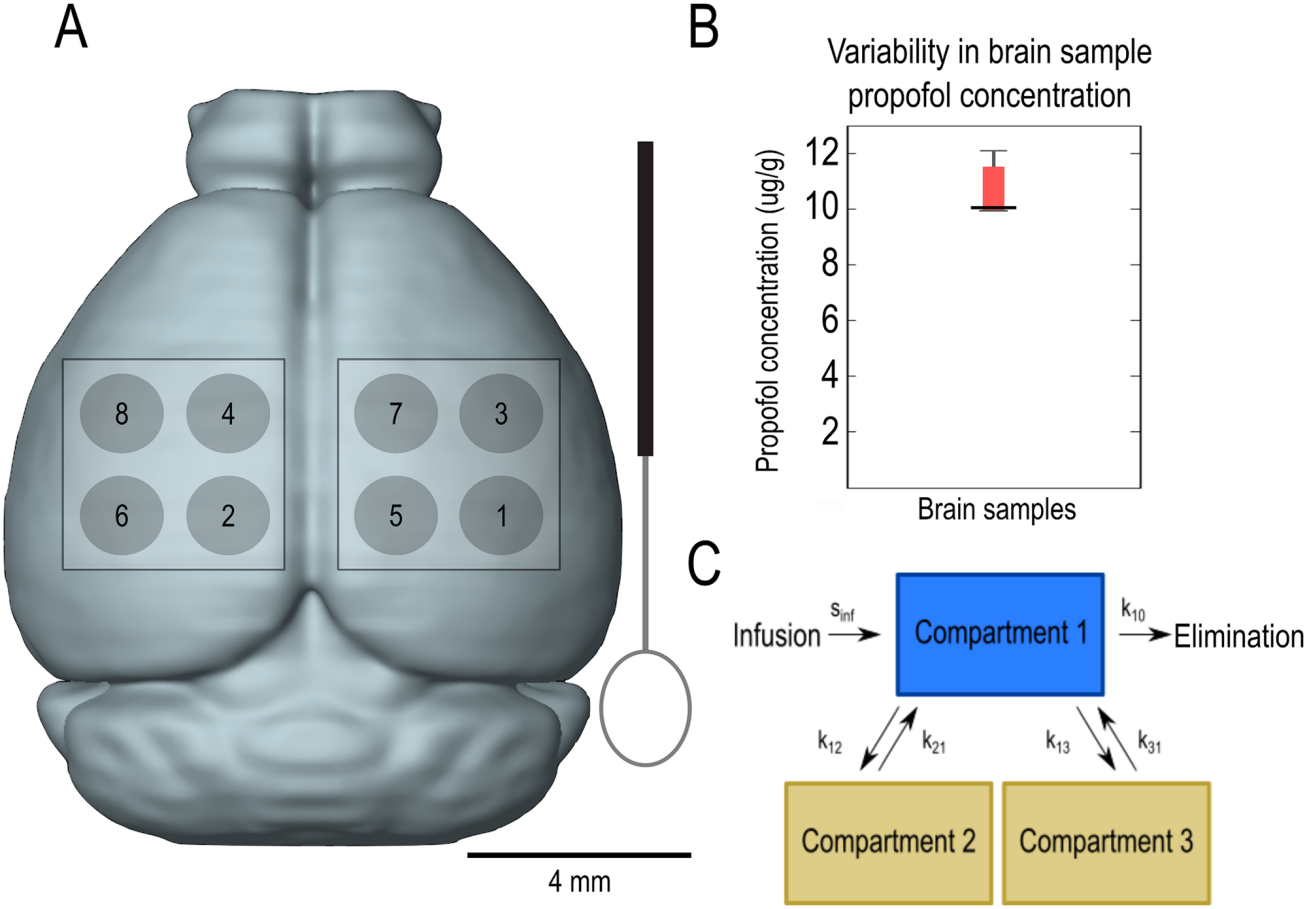
Schematic of sampling procedure. In order to minimize the effect of repeated sampling from the same animal, samples were collected in a consistent order alternating between biopsies shown in (A) along with a drawing of the tool used to obtain brain biopsies. To further establish that this method did not cause significant harm to the surrounding brain tissue, a single large biopsy of brain tissue was removed and sectioned following a 1-hour infusion at 2 mg⋅kg^-1^ ⋅min^-1^. The propofol concentration in each section was analysed. (B) shows the median and interquartile range for the propofol concentration measured in the separate sections of the large biopsy (*n* = 8, median = 10.05, IQR = 1.5). (C) Drug only enters and exits the model through compartment 1. Compartment 1 models blood, compartment 2 models brain, and compartment 3 models all other tissues. *k*_12_, *k*_13_, *k*_21_, and *k*_31_ are the inter-compartmental rate constants, *k*_10_ is the rate of elimination, and *s*_*inf*_ is a constant that was necessary to convert the body weight of the mouse to the theoretical volume of compartment 1.

Cortical biopsies were collected using a custom-made tool with a 2 mm wire loop on the end (Fig 1A) fashioned after a Halasz knife, historically used for spatially restricted brain lesions [30–32]. The tool was lowered just below the surface of the cortex and rotated counter-clockwise and back clockwise to detach a small (~3–8 mg) biopsy of brain tissue. Brain samples were removed in a consistent order (Fig 1A), alternating between sides to minimize the potential effect of tissue trauma on propofol measurements. Care was taken to ensure that there was always an isthmus of non-lesioned brain tissue separating two nearest neighbor biopsy sites. Brain tissue sampling was limited to a maximum of 8 biopsies per animal, due to the small size of the adult mouse brain.

A maximum of two 50–100 μL retro-orbital venopunctures and one terminal transcardiac blood sample were collected. The transcardiac sample was obtained at the end of the experiment to avoid contamination of jugular vein samples by infused propofol. Brain and blood samples were frozen at -80°C until they were analyzed.

Few previous pharmacokinetic studies have directly measured brain propofol concentrations [33]. To determine the experimental measurement variability of brain drug concentration using the planned methodology, an entire hemisphere of the mouse brain was taken at the conclusion of 1-hour propofol infusion (2 mg⋅kg^-1^⋅min^-1^). This large sample was subsequently sectioned into 8 pieces commensurate with individual small biopsies. Propofol measurements were performed independently on each of these pieces (Fig 1B). Differences in the propofol concentration obtained from different portions of the cerebral hemisphere put an upper limit on the spatial variability of propofol concentrations in the brain. We quantify this spatial variability as interquartile range (Fig 1B), which we estimate to be 10–15%. Thus, there do not appear to be dramatic differences in the propofol concentration in different portions of the mouse brain. Propofol concentrations in blood and brain were measured using previously established methodology [34,35].

### Three compartment model

A three compartment model was fitted to the data using MATLAB version R2014a (Mathworks, MA, USA). Compartment 1 models blood, compartment 2 models brain, and compartment 3 models other tissues. Concentrations in compartments 1 and 2 were measured and used to constrain the model fit. Compartment 3 is theoretical. *x*_*i*_(*t*) is defined as the concentration of drug in compartment *i* at time *t*. Rate constants from compartment *i* to compartment *j* are denoted as *k*_*ij*_. *k*_*i*0_ is the elimination rate constant. Drug is eliminated exclusively from compartment 1. The units of rate constants are inverse time. All exchanges are assumed to happen reversibly via compartment 1, with the exception of elimination (Fig 1C). Infusions were administered in units of mg⋅kg^-1^⋅min^-1^. To convert from total body weight of the mouse to the volume of compartment 1, the scaling factor *s*_*inf*_, with units of volume of compartment 1 per kg mouse body weight (L⋅kg^-1^), was used.

The model is given by the following system of linear differential equations where *I*(*t*) is the infusion rate

$$\begin{array}{l} \frac{dx_{1}}{dt}=-\left(k_{12}+k_{13}+k_{10}\right)x_{1}+k_{21}x_{2}+k_{31}x_{3}+\frac{I\left(t\right)}{s_{inf}}\\ \frac{dx_{2}}{dt}=k_{12}x_{1}-k_{21}x_{2}\\ \frac{dx_{3}}{dt}=k_{13}x_{1}-k_{31}x_{3} \end{array}$$ (1)

In order to compute the concentration in each compartment as a function of time, the above system of linear first order differential equations has to be integrated. As *I* is an arbitrary function of time, there is no closed form solution. Thus, this integration was performed numerically using an appropriate ordinary differential equation solver (*ode45*) implemented in MATLAB. To simplify the notation, the above system of equations is combined as follows:

$$\left[\begin{array}{c} x_{1}\\ x_{2}\\ x_{3} \end{array}\right]=P\left(\omega ,I\left(t\right),t\right)$$

where *ω* = {*s*_*inf*,_ *k*_10_, *k*_12_, *k*_13_, *k*_21_, *k*_31_}, *I* is the infusion, *t* is time, and *P* refers to the numerically integrated system Eq 1.

### Fitting procedure

By capitalizing on the experimental design that allows repeated sampling from the same animal an error term was defined as $\varepsilon_{k}(\omega )={\left\|d_{k}-p_{k}\right\|}_{2}^{2}$ where *d*_*k*_ is a set of experimental measurements obtained at times *t*1, *t*2, …, *tn* in animal *k*, and *p*_*k*_ = P(*ω*, *I*(*t*), *t*) is the model defined by parameter set *ω* and evaluated at the same times. That is to say prediction error for each experiment was defined as the *Euclidean* distance between all of the data collected in that experiment and the prediction of the model. Deviations between observed and predicted concentrations in the blood and brain were weighed equally. Overall, the model fits brain concentration better than blood because more brain samples were collected than blood samples. Because it was not always possible to collect identical number of samples in each experiment, *ε*_*k*_ was normalized by the total number of measurements in a given experiment.

In order to find a set of constants, *ω*, that most closely captures the observed kinetics across all experiments, the function $G\equiv \frac{1}{N}\sum_{k=1}^{N}\varepsilon_{k}(\omega )$ was minimized subject to constraint such that all constants in *ω* were non-negative real numbers, where *N* is the total number of animals. The constrained minimization of *G* was implemented using *fmincon* function in MATLAB. As this is a nonlinear problem, local minima are possible. In order to minimize the potential for being trapped in a local minimum, minimization was started in parallel from 100 starting parameter sets. At the end of the minimization, the top 100 solutions obtained during previous round were used as a starting point.

**Fig 2:**
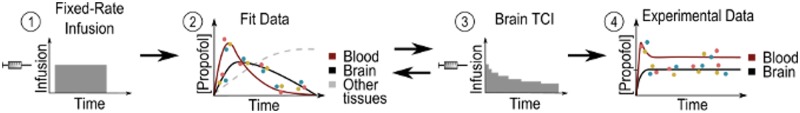
Schematic of model creation methods. The first image represents the initial infusion used, which was delivered at a fixed rate. The second image shows that data collected from these experiments were fit and used to produce estimates of the pharmacokinetic parameters, as described in the methods. Third, these parameter estimates were used to calculate the infusion rate necessary to maintain a target brain concentration for the brain TCI experiments. After each experimental set, accuracy of TCI model was determined and fit was updated to incorporate all experimental findings. The methodology represented in images 2 and 3 was repeated until time-invariant and unbiased target brain concentration of propofol was maintained for at least 1 hour.

The fitting procedure described above was applied iteratively (Fig 2). In the first two experimental paradigms, time invariant infusions were administered. The data collected in these two paradigms were simultaneously fit by a single model. On the basis of these parameters, TCI was implemented, and the resulting brain and blood propofol measurements were used to refine the model fit. This process was continued until satisfactory performance of TCI was attained.

### Implementation of TCI

TCI was implemented in MATLAB by adapting the STANPUMP algorithm.[36] Briefly, as the system in Eq 1 is linear, response to any arbitrary sequence of inputs (*i*.*e*. infusions) can be computed as a convolution of the input signal with the impulse response of the system. Impulse response refers to the modeled response to a unit infusion applied for a single time unit (10 s). Using an algorithm similar to that by Shafer and Gregg, [36] at each time step, an estimate for the next infusion rate was made with the goal of attaining, then maintaining the targeted brain concentration. This estimate was iteratively refined, by convolving the system with the impulse response, until the predicted brain concentration was within acceptable deviation (5%) from the target.

### Electrophysiology and preprocessing

Adult male mice (20–25 g), were used for electrophysiology. After a craniotomy and jugular cannulation (see above), a silicone multielectrode array (Neuronexus: E64-500- 20–60) was positioned epidurally. Signals over the somatosensory cortex were recorded relative to a reference screw placed into contralateral skull, sampled at 3030.3 Hz (Cheetah 64, Neuralynx) and recorded onto a hard drive for post hoc analysis. Upon starting propofol infusion, isoflurane was turned off. Signal collection was initiated 20 minutes after start of propofol infusion. Target brain concentration of 10 μg⋅g^-1^ and 15 μg⋅g^-1^ were used.

### Statistical analysis

In order to determine whether the concentration of propofol in the final set of mouse brain samples varied over time, a nonparametric Wilcoxon test was used to compare concentrations collected from the first and final 20 minutes of a one-hour infusion. In addition, the best-fit line was calculated for brain sample propofol concentrations. The slope of this line, *b*, was determined and its standard error was calculated using the following equation

$$S.E.=\frac{S\left(c\right)}{\left(n-2\right)S\left(t\right)}$$

where $S(x)=\sqrt{\sum {\left(x_{i}-\overset{-}{x}\right)}^{2}}$, *c* is concentration, *t* is time, and *n* is the total number of measurements.

To further assure the temporal stability of the fit, the autocorrelations of the residual were evaluated for the final TCI model using Ljung-Box Q-test (*lbqtest* in Matlab).[37] In order to test whether or not there was a bias in our model the median performance error and median absolute performance error were computed using the following equation

$$PE=\frac{C_{m}-C_{p}}{C_{p}}\times 100$$

where *PE* is the performance error of the model, and *C*_*m*_ and *C*_*p*_ are the measured and predicted brain propofol concentrations, respectively.[38,39] The median performance error is the median value of performance errors, and median absolute performance error is the median of the absolute values of these errors.

## Results

**Fig 3:**
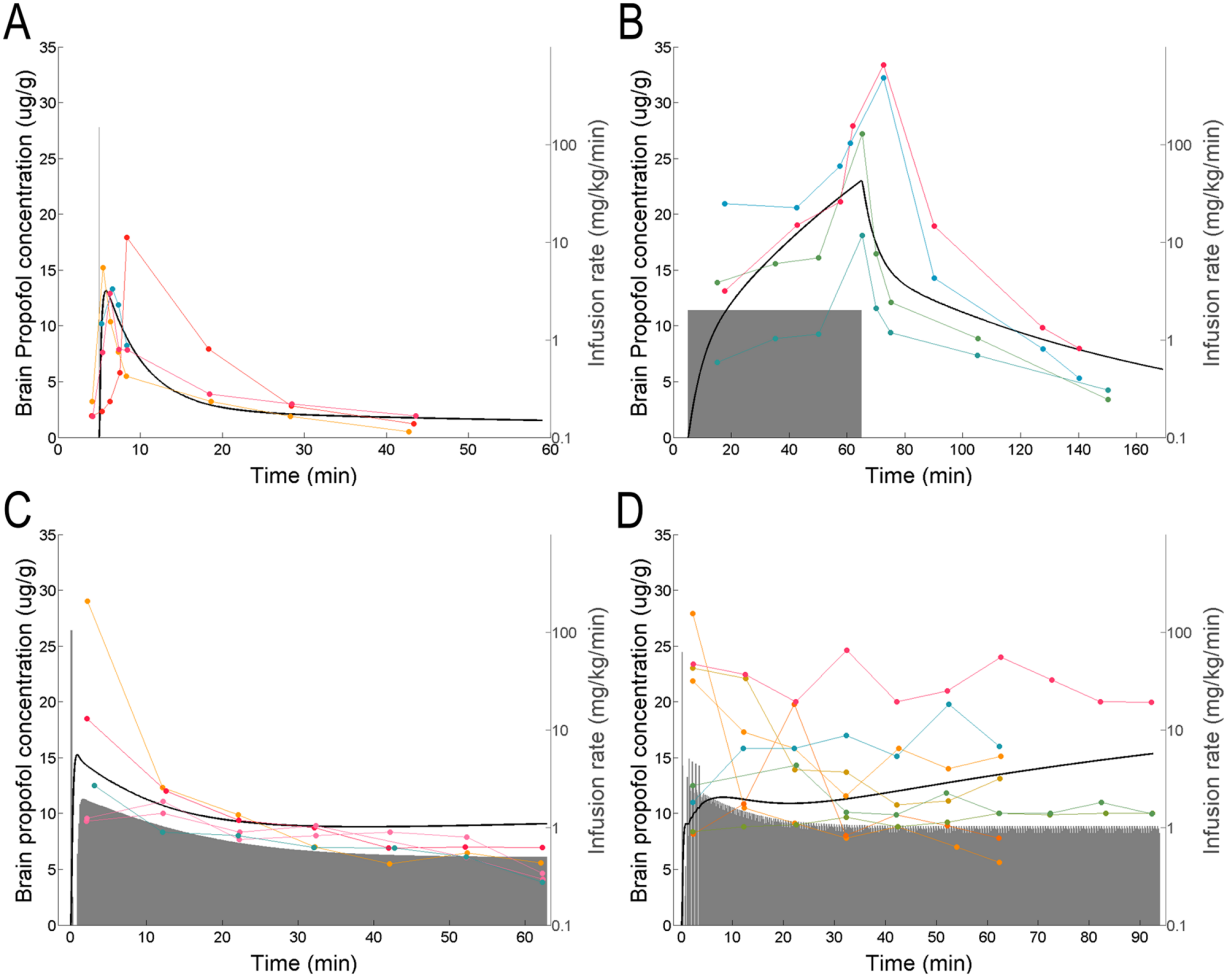
Brain propofol concentration data used for model fitting. Brain propofol concentration measured and fitted for all experiments used in model creation. In each graph, the shaded grey area shows the infusion rate in mg⋅kg^-1^⋅min^-1^ used in each experiment. These rate data are plotted on a log scale displayed on the right y-axis. Heavy black lines show the propofol concentration predicted in the brain tissue in response to the infusion used in that experimental set according to the final set of pharmacokinetic rate constants obtained after fitting all four experimental paradigms. Connected points indicate the propofol concentration measured in the brain tissue samples from a single subject. (A) and (B) show the simple infusions used. (A) 150 mg⋅kg^-1^⋅min^-1^ for 6 seconds. (B) 2 mg⋅kg^-1^⋅min^-1^ for 1 hour. (C) and (D) show the infusions resulting from the first and second attempts at achieving brain TCI, both targeting 10 μg/g in the brain tissue.

**Fig 4:**
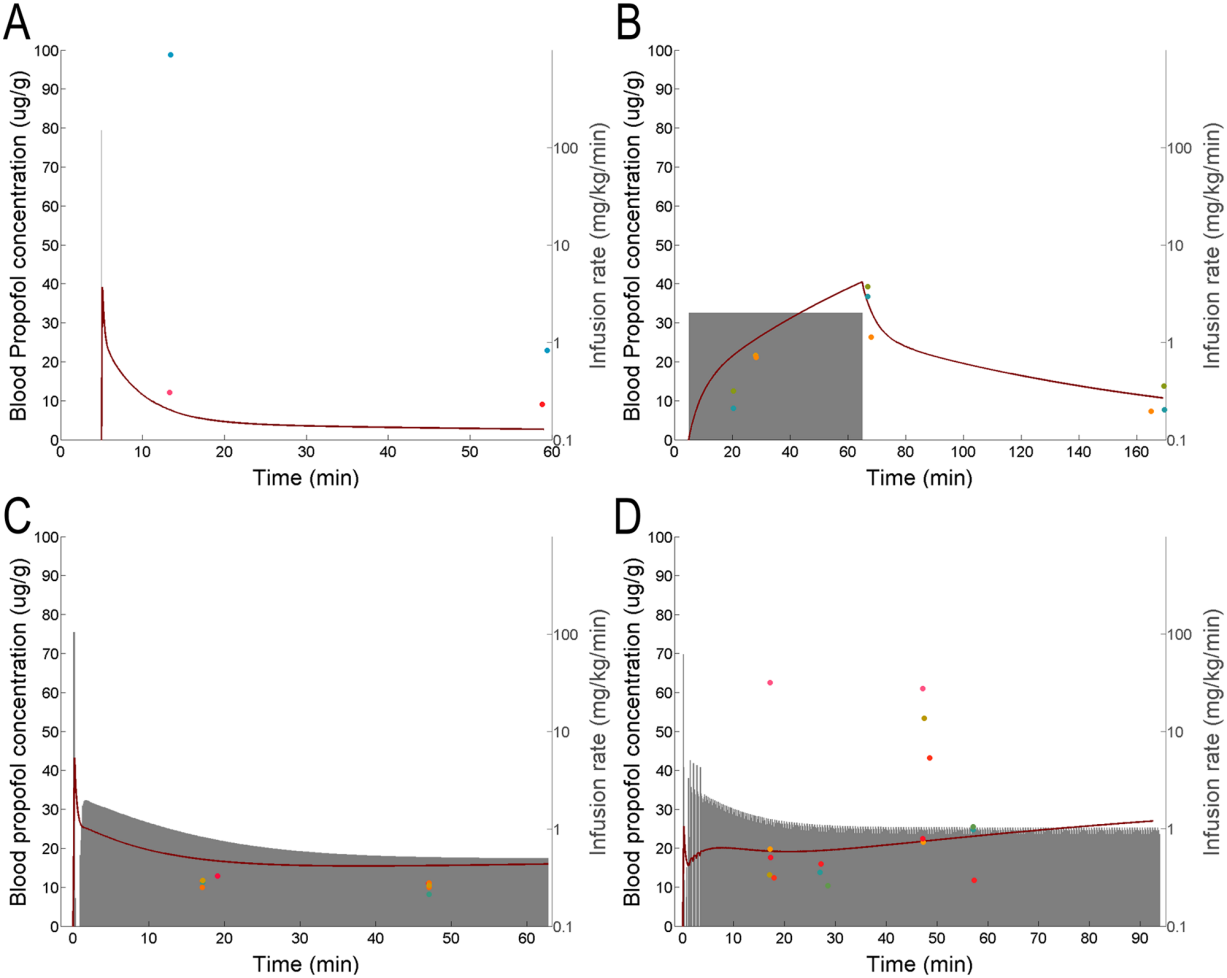
Blood propofol concentration data used for model fitting. Blood propofol concentrations measured from the same experiments represented in Fig 3. As in Fig 3, the shaded grey area shows the infusion rate in mg⋅kg^-1^⋅min^-1^ used in each set of experiments. Heavy red lines show the propofol concentration predicted in the blood. Connected points indicate the propofol concentration measured in the blood of a single subject. (A) and (B) show the simple infusions used. (A) 150 mg⋅kg^-1^⋅min^-1^ for 6 seconds. (B) 2 mg⋅kg^-1^⋅min^-1^ for 1 hour. (C) and (D) show the infusions resulting from the first and second attempts at achieving brain TCI, both targeting 10 μg/g in the brain.

After establishing the consistency of measurements across different brain regions (Fig 1B), initial experiments were performed using a bolus (150 mg⋅kg^-1^⋅min^-1^ infusion lasting 6 seconds *i*.*e*. 15 mg⋅kg^-1^ bolus) (Figs 3A and 4A, *n* = 4 animals) and a 1-hour fixed infusion at 2 mg⋅kg^-1^⋅min^-1^ (Figs 3B and 4B, *n* = 4 animals). The first generation model was fit to the data from these 8 experiments. This yielded an initial set of model parameters that were iteratively refined. To refine model parameters, we computed the time-varying infusion rate necessary to maintain constant brain concentration. For this purpose we chose 10 μg⋅g^-1^ (brain concentration). This concentration was chosen because it was within the range of concentrations observed in the initial experiments (Fig 3A and 3B). TCI using the initial estimates of model parameters gave rise to a slowly decreasing brain propofol concentration (Fig 3C, *n* = 5 animals). Thus, the model parameters were refined by fitting the data from the first 3 sets of experiments (*n* = 13 animals). This model gave rise to the data shown in Figs 3D and 4D. While second generation of the model fixed the slow downward drift, it yielded consistent slight overshoot in the measured brain concentration relative to the target. Thus, a third generation of the model was created by fitting simultaneously all four infusion paradigms (241 samples collected from 21 animals: 193 brain, 48 blood). The concentrations predicted by this third generation of the model for brain and blood are shown as thick black and red lines respectively in Figs 3 and 4.

**Fig 5:**
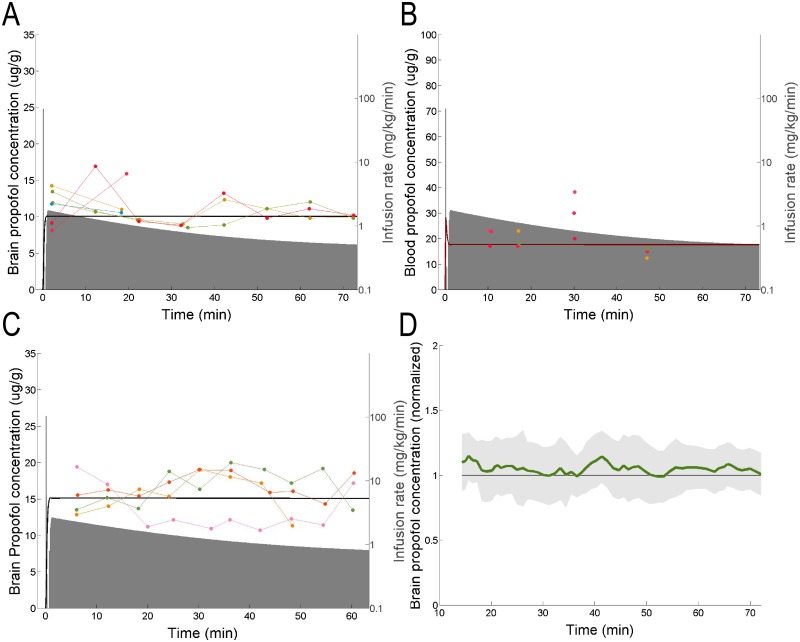
Model validation data. The infusion (grey shaded area) was computed to target brain concentration of 10 μg⋅g^-1^ (A and B) or 15 μg⋅g^-1^ (C). Predicted propofol concentration in the brain (A and C) and blood (B) are shown by thick black and red lines, respectively. Measured propofol concentration in the brain (A and C) and blood (B) are shown as points. Points are colour coded by subject. The data from panels A and C were replotted in (D) as the moving average and standard deviation of the normalized propofol concentration in brain tissue, relative to the target concentration.

A good way to evaluate the validity of a model is to determine whether the model is capable of predicting experimental results to which it was not explicitly fit [40]. For model validation, samples were collected from 10 additional animals (68 brain, 12 blood). Note that in contrast to the data shown in Figs 3 and 4, data presented in Fig 5 were not used to fit the model. Thus, results in Fig 5 are a *bona fide* prediction of both brain (Fig 5A and 5C) and blood (Fig 5B).

In Fig 5A and 5B we targeted 10 μg⋅g^-1^ brain concentration. To make sure that the model can be used to target a different brain concentration, in Fig 5C we used 15 μg⋅g^-1^.

To quantify the degree to which TCI gave rise a constant brain propofol concentration, we compared measurements obtained during the first 20 minutes (*n* = 12 measurements for Fig 5A; *n* = 11 for Fig 5C) to those obtained in the last 20 minutes of infusion (*n* = 12 measurements for Fig 5A; *n* = 14 for Fig 5C) (*p* = 0.28, Fig 5A; *p* = 0.93, Fig 5C, Wilcoxon). No statistically significant drift in brain propofol concentration was detected. Thus, we are unable to detect any significant change in the brain concentration of propofol over one hour of infusion. Furthermore, the slope of a line fitted to the data in Fig 5A was -3x10^-4^ (μg⋅g^-1^⋅s^-1^). The 95% confidence interval on this slope was [-9x10^-4^, 2x10^-4^]. The slope of the line in Fig 5C was -2x10^-4^ (μg⋅g^-1^⋅s^-1^) with a 95% confidence interval [-6x10^-4^, 2x10^-4^]. Thus, linear fitting is also unable to detect significant drift in propofol concentration over time. No evidence of autocorrelation in the residuals (*p* > 0.05, Fig 5A and 5C, Ljung-Box Q-test) was found. Thus, the null hypothesis that the residuals are random deviations from the prediction cannot be rejected. Results were unbiased yielding brain concentration 10.95 ± 2.1 μg⋅g^-1^ (mean ± standard deviation) in Fig 5A and 15.6 ± 2.8 μg⋅g^-1^ (mean ± standard deviation) in Fig 5C. Furthermore, the median performance error of the model was 2% with a 95% confidence interval of [-2%, 7%]. As the 95% confidence interval includes 0, no statistically significant bias can be detected. To combine the results from validation cohorts targeting 10 μg⋅g^-1^ and 15 μg⋅g^-1^, we normalized the measured propofol concentration by the target concentration. We then computed the mean and the standard deviation of the data in sliding windows (window length 10 consecutive samples stepped by 1 sample). The results are shown in Fig 5D. The measured brain concentration was within less than one standard deviation of the target concentration for all time points across two independent sets of experiments. Thus, it was unlikely that increasing the number of experiments will significantly alter the model parameters. Given the results of our statistical testing, the third set of rate constants were taken as our final model parameters (Table 1).

**Table 1:** Constants of final model.

Direction of Diffusion	*k*_*ij*_ (min^-1^)
Blood to brain, *k*_12_	1.55
Brain to blood, *k*_21_	2.71
Blood to other tissues, *k*_13_	0.22
Other tissues to blood, *k*_31_	0.04
Elimination, *k*_10_	0.07
Scaling constant for infusion (L⋅kg^-1^), *s*_*inf*_	0.35

Rate constants (*k*_*ij*_) denote drug movement between compartments. The scaling constant *s*_*inf*_ expresses litres of compartment 1 per kg of total body weight.

**Fig 6:**
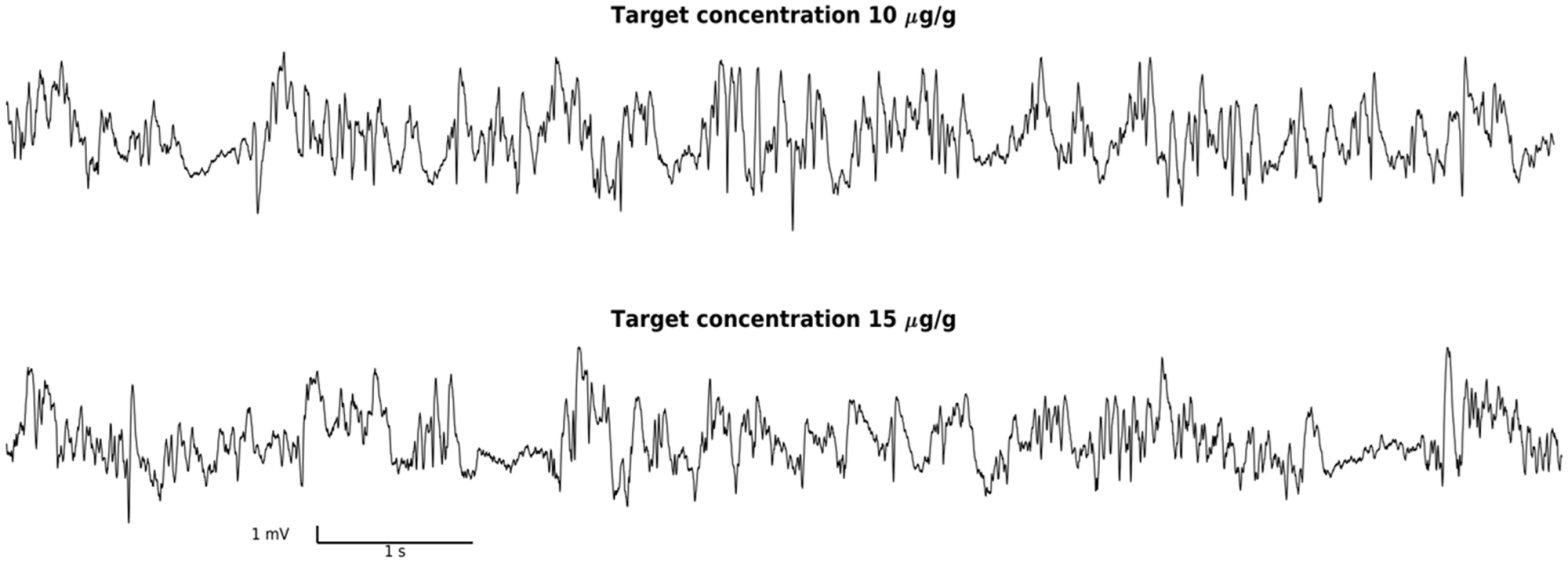
Electrocorticographic verification of anesthetic depth. Each trace represents a 10 second segment of spontaneous ECoG recorded over the mouse somatosensory cortex while the mouse is receiving TCI propofol. This data was collected beginning 20 minutes into a 30 minute recording. The target brain concentration of propofol is 10 μg⋅g^-1^ in the top trace (A). This trace shows that the alpha frequency is prominent from 4–7 seconds. In contrast, the target brain concentration in the bottom is 15 μg⋅g^-1^ (B), during which a deeper anesthetic state is illustrated by burst suppression.

To determine that the targeted propofol concentrations produce neurophysiologically-defined state of anesthesia, we recorded local field potentials (LFPs) extradurally over the somatosensory cortex while targeting both 10 μg⋅g^-1^ and 15 μg⋅g^-1^. In these experiments isoflurane was turned off 20 minutes prior to recording. This is sufficient of isoflurane washout from mouse brain [5]. Thus, LFP recordings (Fig 6) confirm that targeted propofol concentrations are sufficient to produce anesthesia on their own. Furthermore, increasing from 10 to 15 μg⋅g^-1^ resulted in more slowing in the LFP, consistent with increasing anesthetic depth.

## Discussion

In order to connect the molecular and network level effects of propofol, it is necessary to precisely control brain propofol concentration in the brain. The model presented here is capable of accomplishing this rather well.

All of the software developed as a part of this project was written in MATLAB. With minor adjustments, MATLAB can be used with a number of commercially available syringe pumps, if the serial interface is known. The MATLAB code developed herein will be made freely available upon request. All relevant code is available from the Zenodo repository at the following DOI: 10.5281/zenodo.1205136. While there is some propofol pharmacokinetic data available in rats [34] and rabbits [41] very little is available in mice [42]. Yet, mice offer significant advantages as a model system for neuroscientists because of wide availability of genetic tools for interrogating and manipulating neuronal activity. Thus, development of a tool capable of precise delivery of propofol to the brain of mice is likely to prove useful.

One of the technical innovations introduced herein is the serial sampling of brain propofol concentration. As a result, the TCI based on the model gives rise to constant brain concentration not just across different individuals, but also across time in the same individual. The success of this method is predicated on the fact that brain propofol concentration does not differ dramatically between different brain regions (Fig 1B). In order to perform serial brain sampling while avoiding responsiveness to painful stimuli, we used a trace amount of isoflurane (0.2%) during propofol infusions. This is a sub-anesthetic dose– 100 percent of mice maintain their righting reflex at 0.63% isoflurane [5]. Even significantly higher isoflurane concentrations (1.5%) do not disrupt hemodynamics appreciably in mice [29]. Thus, it is unlikely that 0.2% isoflurane significantly alters propofol distribution into and elimination out of the brain. In a separate set of experiments, we confirm using direct recordings of neuronal activity that the targeted concentration produces the expected slowing of the electroencephalogram.

Early iterations of the experiments were performed with tail vein rather than jugular vein cannulation. Prolonged infusions via the tail vein were found to be highly unreliable. Therefore, no data from tail vein administration experiments were used for model fitting.

Both existing PK and closed loop anesthesia delivery systems depend on the existence of a fixed relationship between drug concentration and its effect on brain activity. The present study, is free from this assumption. Rather than inferring concentration in a hypothetical “effect site” compartment on the basis of brain activity, the concentration of the anesthetic agent in the brain was measured directly. Fixing the brain propofol concentration at a desired level will allow for the investigation of effects of propofol on neuronal dynamics *in vivo*.
